# Narrow-Band Deep-Blue Emission and Superior Thermal Stability of Fluoroaluminate Phosphor Based on Tungsten Bronze-Type Mineral Structure

**DOI:** 10.3390/ma16145053

**Published:** 2023-07-17

**Authors:** Rui Lu, Jianfeng Sun

**Affiliations:** 1 School of Materials Science and Engineering, Hebei University of Technology, Tianjin 300130, China; m13230321575@163.com; 2 Key Laboratory of Special Functional Materials for Ecological Environment and Information, Hebei University of Technology, Ministry of Education, Tianjin 300130, China

**Keywords:** tungsten bronze-type structure, KCaAl_2_F_9_:Eu^2+^, narrow-band deep-blue emission, full-spectrum lighting, warm white light-emitting diodes

## Abstract

Screening novel narrow-band phosphors inspired by natural mineral structures is urgently demanded for improving the performance of phosphor-converted light-emitting diodes. In this work, a novel narrow-band deep-blue-emitting tungsten bronze-type KCaAl_2_F_9_:Eu^2+^ phosphor with superior thermal stability is successfully synthesized. Structural analysis shows that the representative KCaAl_2_F_9_:0.013Eu^2+^ phosphor crystallizes in an orthorhombic space group *C*222_1_ with a rigid network. The rigid [AlF_6_]^3−^ octahedrons are linked together by sharing corners to build endless [AlF_6_]^3−^_∞_ chains, further stacking with each other in a highly cross–linked way to establish the rigid network of the KCaAl_2_F_9_ host. Benefiting from the rigid microenvironment, the developed phosphor not only shows a narrow-band deep-blue emission with a full width at half maximum of 45 nm and a high color purity of 92%, but it also exhibits the superior thermal stability with an emission loss of only 10% at 423 K, demonstrating its application potential in bridging the deep-blue spectral cavity toward sunlight-like full-spectrum lighting. In addition, the concentration/temperature quenching behaviors of KCaAl_2_F_9_:Eu^2+^ phosphor are systematically investigated. By revealing the specific structure–property relationship of tungsten bronze-type KCaAl_2_F_9_:Eu^2+^ phosphor, the present study provides a significant guide for identifying the novel narrow-band deep-blue-emitting component applicable to full-spectrum warm white light-emitting diode devices.

## 1. Introduction

Phosphor-converted light-emitting diodes (pc-LEDs) have been recognized as a key enabler for the emerging artificial light source used in modern technologies such as lighting, display, and plant cultivation, as well as biomedical imaging and illumination owing to their superiorities of high efficiency, high level of engineering control, long service life, and environmental friendliness [1,2]. For pc-white LEDs (pc-WLEDs) known as the next-generation lighting source, the significant performance indexes such as correlated color temperature (CCT) and color rendering index (CRI) dominated by spectral energy distribution play an essential role in achieving the desirable white light. Nevertheless, the modern commercial pc-WLED architecture consisting of blue InGaN LED chip and yellow-emitting Y_3_Al_5_O_12_:Ce^3+^ phosphor always suffers from high CCT (*T*_c_ > 4500 K) and poor CRI (*R*_a_ < 75) owing to the insufficient red spectral component in emission [3]. On the other hand, the severe blue-light hazard associated with the unconverted sharp blue light from blue InGaN LED chips can further disrupt the human circadian rhythm and metabolism because the spectral curve of the InGaN LED chip overlaps considerably with the maximum of the wavelength-dependent melatonin suppression curve, possibly causing related health problems such as mood disorders, insomnia, and cataract formation after chronic blue-light exposure [4]. As an alternative, the emerging strategy for implementing full-spectrum warm white emission in WLEDs by utilizing the combination of red, green, and blue phosphors and ultraviolet (UV)-pumped LED chips has recently been proposed [5]. This scheme can effectively avoid the blue-light hazard of conventional pc-WLEDs. However, the emission wavelengths of currently available blue phosphors are commonly located in the long-wavelength (440–480 nm) region and result in the unneglected spectral cavities in the deep-blue (400–430 nm) and blue-cyan (480–520 nm) regions with an inevitable reduction in the CRI of corresponding warm WLEDs, which is considered to be the bottleneck for achieving full-spectrum warm white lighting aimed at simulating natural sunlight [6]. Until recently, several blue-cyan-emitting phosphors have been well developed as the components in warm WLEDs to enhance the color quality via filling the blue-cyan spectral cavity, such as Ca_2_(Lu,Y)Hf_2_Al_3_O_12_:Ce^3+^ [7,8], BaScO_2_F:Eu^2+^ [9] and Rb_2_CaPO_4_F:Eu^2+^ [10]. Nonetheless, despite the few emerging deep-blue phosphors, typically Ln_2_Bi_2_O_7_:Bi^3+^ (Ln = Gd, Y, Lu, Sc), the deep-blue-emitting phosphors bridging violet and blue spectral regions still have been rarely studied, which remains a challenging task for realizing the sunlight-like full-spectrum lighting with high CRI in warm WLEDs [6].

A design strategy based on natural mineral-type structures is conducive to discovering novel phosphors owing to their facile structure–property related regulation resulting from the definite relationship between mineral structure and luminescence property [11]. Until recently, most of the emerging narrow-band blue-emitting silicate phosphors are based on the original UCr_4_C_4_-type structural prototype, such as RbNa_3_[Li_3_SiO_4_]_4_:Eu^2+^ [12], RbNa_2_K[Li_3_SiO_4_]_4_:Eu^2+^ [13], RbKLi_2_[Li_3_SiO_4_]_4_:Eu^2+^ [14], and Na_0.5_K_0.5_[Li_3_SiO_4_]:Eu^2+^ [15] (Table 1), all of which are featured with a rigid structure with a high degree of condensation composed of edge- and vertex-sharing [SiO_4_]^4−^ and [LiO_4_]^7−^ tetrahedrons.

This structural characteristic endows them with the ability to decrease the emission bandwidth and resist the thermally induced lattice vibration to reduce the thermally activated phonons, which is favorable for their narrow-band emissions and high thermal stabilities [23]. Other recent studies have also demonstrated that some non-UCr_4_C_4_-type mineral structures can be utilized as prototype models to design the novel narrow-band phosphors. For example, the perovskite-type Cs_4_Mg_3_CaF_12_:Eu^2+^ and K_2_BaPO_4_F:Eu^2+^ phosphors exhibit the narrow-band blue-emitting feature with favorable thermal stability (*λ*_max_ = 474 nm, full width at half maximum (FWHM) = 55 nm, *I*_473K_/*I*_298K_ = 80% for Cs_4_Mg_3_CaF_12_:Eu^2+^; *λ*_max_ = 439 nm, FWHM = 25 nm, *I*_423K_/*I*_298K_ = 98% for K_2_BaPO_4_F:Eu^2+^) [16,17]. Nonetheless, the spectral energies of the abovementioned phosphors mainly distribute in the long-wavelength (430–490 nm) region and are unable to compensate for the spectral cavity in the deep-blue region. Until now, utilizing the design strategy of mineral-type structural prototypes to explore novel deep-blue-emitting phosphors is still significant and urgent for improving the CRI toward the sunlight-like full-spectrum lighting applicable to warm WLED devices.

By screening various non-UCr_4_C_4_-type structural prototypes, we found that the tungsten bronze-type (TB-type) structure with the general chemical formula of (A1)_4_(A2)_2_C_4_M_10×30_ shows a great compositional flexibility owing to the multiple cation sites based on its unique structural framework consisting of corner-sharing [MX_6_] octahedrons, which exhibit potential to develop novel phosphors by modulating the local coordination environments of dopant sites through structural unit substitution [24]. Until recently, several TB-type oxide ceramics or phosphors have been systematically investigated, such as (Sr,Ba)Nb_2_O_6_:Eu^3+^ (*λ*_max_ = 621 nm) [24], BaTa_2_O_6_:Sm^3+^ (*λ*_max_ = 597 nm) [25], Sr_5_YTi_3_Nb_7_O_30_:Eu^3+^ (*λ*_max_ = 613 nm) [26], and Sr_1.90_Ca_0.15_Na_0.9_Nb_5_O_15_:Eu^3+^ (*λ*_max_ = 617 nm) [27], some of which can be utilized as the red component applicable for WLEDs. Nevertheless, until recently few studies have been dedicated to developing novel narrow-band deep-blue-emitting phosphors based on the TB-type structural prototype. By thoroughly screening the known TB-type compounds, we consider the KCaAl_2_F_9_ (KCAF) as a favorable host which benefits the induction of narrow-band luminescence due to its rigid structural framework composed of highly cross–linked infinite [AlF_6_]_∞_ chains [28]. In the present study, we first utilize TB-type KCAF as the structural prototype to develop a novel narrow-band deep-blue-emitting KCAF:Eu^2+^ phosphor. Rietveld structural refinements reveal that the Eu^2+^ ions occupy two types of crystallographic Ca^2+^ sites in the [Ca(1)F_6_]^4−^ and [Ca(2)F_6_]^4−^ octahedrons of the KCAF host lattice, thereby resulting in favorable narrow-band deep-blue luminescence (*λ*_max_ = 400 nm, FWHM = 45 nm) with a wide color gamut (87% of the National Television System Committee (NTSC) standard), excellent color purity (92%), and thermal stability (*I*_423K_/*I*_298K_ = 90%), which surpasses the currently emerging deep-blue-emitting phosphors and most of the aforementioned UCr_4_C_4_-type blue-emitting silicate phosphors. In addition, the origins of narrow-band deep-blue luminescence and thermal quenching behavior are systematically investigated on the basis of the structural characteristics of the TB-type KCAF host lattice. By further revealing the specific structure–property relationship of KCAF:Eu^2+^, our present study provides a potential guiding significance for identifying the novel narrow-band deep-blue-emitting phosphor applicable in bridging the deep-blue spectral cavity toward sunlight-like full-spectrum lighting.

## 2. Experimental Section

### 2.1. Material Synthesis

The TB-type KCAF:*x*Eu^2+^ samples (*x* = 0–0.013) were prepared through a solid-state reaction. The stoichiometric amounts of starting raw materials of KF (analytical reagent, (A.R.); Tianjin Sailboats Chemical Reagent Co., Ltd., Tianjin, China), CaF_2_ (A.R.; Tianjin Sailboats Chemical Reagent Co., Ltd., Tianjin, China), AlF_3_ (A.R.; Tianjin Bodi Chemical Co., Ltd., Tianjin, China), and Eu_2_O_3_ (99.99%; Jining Tianyi New Materials Co., Ltd., Jining, China) were weighed and mixed by grinding in an agate mortar for 40 min. Then the obtained well-mixed mixtures were placed into alumina crucibles, heated at a rate of 1.5 K·min^−1^ in a furnace under the reducing atmosphere of CO and calcined at 973 K for 7 h. The resulting samples were reground thoroughly after natural annealing in the furnace for 10 h for subsequent characterization.

### 2.2. Material Characterization

The powder X-ray diffraction (XRD) data of the samples for phase analysis were collected by a Bruker D8 Discover X-ray diffractometer (Bruker, Germany) using Cu Kα radiation (λ = 1.5418 Å) over 2θ range of 5–80°. The high-quality diffraction data for Rietveld refinement were recorded by the same equipment with a slow scanning speed of 0.3°·min^−1^ over 2θ range of 5–130°. The Rietveld refinement was performed using the general structure analysis system program [29]. The morphology and microstructure features of the representative sample were characterized by a field emission scanning electron microscope (FE-SEM, FEI Quanta-450 FEG, FEI, the United States) and transmission electron microscopy (TEM, JEM-2010, JEOL, Japan), and the elemental composition and distribution were observed by energy-dispersive X-ray spectrometry (EDS) attached to the SEM. The electronic band structure of the KCAF host was investigated through the first-principle calculation based on density functional theory (DFT), performed with the Cambridge Serial Total Energy Package mode [30]. The electrons of K-3p 4s, Ca-3p 4s 3d, Al-3s 3p, and F-2s 2p orbitals were treated as valence electrons, and the interaction of the electrons with ion cores was described by the norm-conserving pseudo-potentials. In the calculation process of exchange-correlation potential, the generalized gradient approximation with the Perdew–Burke–Ernzerhof functional was employed [31]. The plane-wave energy cut-off was determined to be 940 eV. The Monkhorst–Pack scheme for the host was chosen as 8 × 8 × 8 in the Brillouin Zone. The diffuse reflection spectra were obtained with a UV–visible spectrophotometer (TU-1901, PERSEE, China) with the BaSO_4_ serving as a reference. The X-ray photoelectron spectroscopy (XPS) spectra were recorded using an X-ray photoelectron spectrometer (PHI 5000 VersaProbe, PHI, Japan) to identify the valence of individual elements. The photoluminescence (PL) spectra and photoluminescence excitation (PLE) spectra were collected by a fluorescence spectrometer (FS5, Edinburgh Instruments, the United Kingdom) equipped with a 150 W Xe lamp. The temperature-dependent PL spectra were recorded on the same spectrometer combined with a self-regulating thermal accessory. The fluorescence decay curves and quantum efficiency (QE) were measured using a spectrofluorometer (JOBIN YVON FL3-22, HORIBA, the United States) with excitation at 310 nm and a monitored wavelength of 400 nm. All the above measurements were conducted at room temperature (RT).

## 3. Results and Discussion

### 3.1. Phase Identification and Crystal Structure

**Figure 1:**
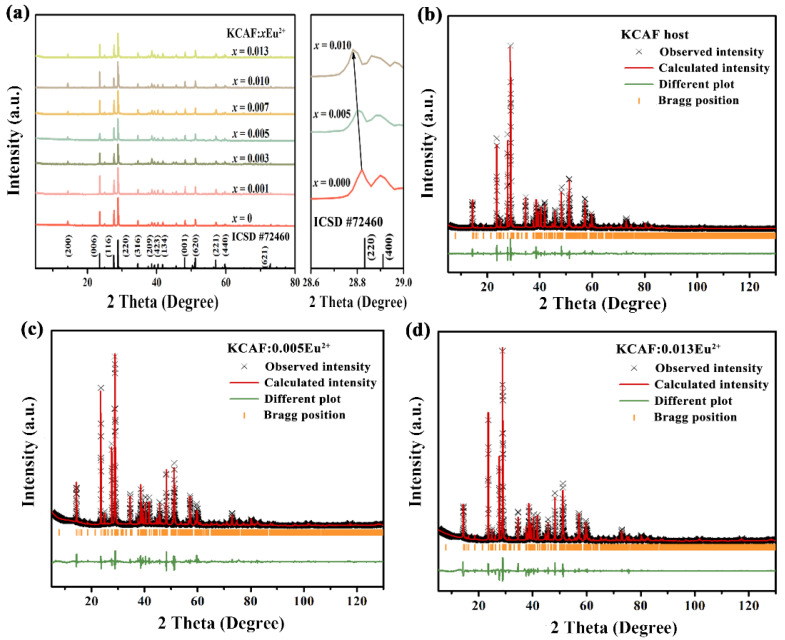
(**a**) XRD patterns for the TB-type KCAF:*x*Eu^2+^ (*x* = 0–0.013) samples and the enlarged main diffraction peak of the KCAF host and representative Eu^2+^-doped KCAF phosphors. (**b**–**d**) Rietveld refinement for the XRD data of the KCAF host, KCAF:0.05Eu^2+^, and KCAF:0.013Eu^2+^ phosphors.

XRD patterns of all the as-prepared TB-type KCAF:*x*Eu^2+^ (*x* = 0–0.013) samples were first given to verify their phase purity. As shown in Figure 1a, all the obtained diffraction peaks can be indexed with those of standard card (ICSD #72460, KCAF), and no secondary phases are detected for the samples with different Eu^2+^ doping content, which suggests that the doped Eu^2+^ ions are completely dissolved into the TB-type KCAF lattice without changing the phase structure.

The magnified XRD patterns in Figure 1a display a minute shift of the diffraction peak of (220) crystallographic plane towards the small-angle direction with increasing Eu^2+^ concentration, implying the lattice expansion of the KCAF host due to the radius difference between Eu^2+^ ions and substituted host cations [32,33]. The substitution of Eu^2+^ ions in the KCAF host lattice can be preliminarily inferred according to the radius percentage difference (*D*_r_) calculated as follows [34]:

(1) $$D_{r}=\left|\frac{R_{m}\left(\mathrm{CN}\right)-R_{d}\left(\mathrm{CN}\right)}{R_{m}\left(\mathrm{CN}\right)}\right|\times 100\%$$

in which *R*_m_(CN) and *R*_d_(CN) are the radii of the host cations and the dopant ions, respectively, and CN is the coordination number. For the KCAF:Eu^2+^ phosphor, *R*_K_^+^(10) = 1.590 Å, *R*_Ca_^2+^(6) = 1.000 Å, *R*_Al_^3+^(6) = 0.535 Å, *R*_Eu_^2+^(10) = 1.350 Å, and *R*_Eu_^2+^(6) = 1.170 Å [33]. For the substituted cations in a specific phosphor, it is accepted that the cationic radii difference between the dopant and substituted ions should be within 30% [34]. Based on Equation (1), the values of *D*_rK_^+^(10), *D*_rCa_^2+^(6), and *D*_rAl_^3+^(6) are calculated to be 15.1%, 17.0%, and 118.7%, respectively, which implies the possibility of Eu^2+^ ions occupying K^+^ and Ca^2+^ sites rather than Al^3+^ site. To further investigate the occupancies of Eu^2+^ ions in the KCAF host lattice, the Rietveld refinement patterns of the KCAF host, KCAF:0.005Eu^2+^, and KCAF:0.013Eu^2+^ phosphors together with their unit cell parameters are shown in Figure 1b–d, and Table 2.

One can see that the well-fitted, refined XRD patterns are identical to its standard XRD card (ICSD #72460) without any impurity phase. As shown in Table 2, all samples crystallize in the TB-type orthorhombic system with a space group of *C*222_1_. The refined cell parameters of KCAF:0.005Eu^2+^ (*a* = 12.329(8) Å, *b* = 7.154(6) Å, *c* = 22.619(3) Å, and *V* = 1995.356(7) Å^3^) and KCAF:0.013Eu^2+^ (*a* = 12.331(7) Å, *b* = 7.156(3) Å, *c* = 22.623(8) Å, and *V* = 1996.535(5) Å^3^) are similar to those of the KCAF host (*a* = 12.329(6) Å, *b* = 7.152(7) Å, *c* = 22.618(8) Å, and *V* = 1994.750(4) Å^3^), which also demonstrates the pure phase characteristics of Eu^2+^-doped KCAF phosphors.

**Figure 2:**
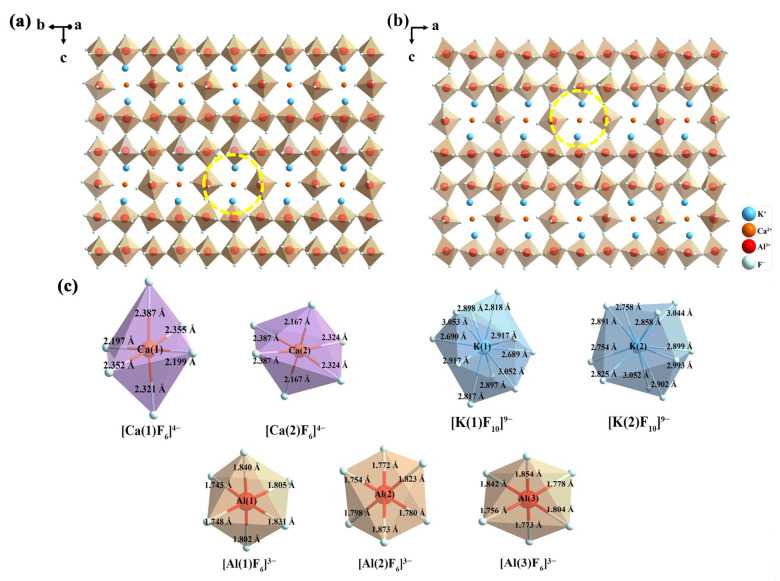
(**a**,**b**) Structure overview of the KCAF host along the [110] and [010] directions. (**c**) The coordination environments of Ca^2+^, K^+^, and Al^3+^ cations in the TB-type KCAF host.

The atomic positions and site occupancies of KCAF:0.005Eu^2+^ and KCAF:0.013Eu^2+^ are further summarized in Table S1. By preliminarily assuming that the sums of occupancy probabilities of occ(Eu(1)^2+^)+occ(Ca(1)^2+^) and occ(Eu(1)^2+^)+occ(Ca(2)^2+^) are both equal to 1, the final reliability parameters of refinement converge to *R*_wp_ = 6.57% and *R*_p_ = 4.25% for KCAF:0.005Eu^2+^ phosphor and *R*_wp_ = 6.78% and *R*_p_ = 4.37% for KCAF:0.013Eu^2+^ phosphor, thus confirming the substitution of the smaller Ca^2+^ cations by the larger Eu^2+^ ions in the KCAF host lattice. As further shown in Figure S1a,b, the refined cell volumes of KCAF:*x*Eu^2+^ (*x* = 0, 0.005, 0.013) slightly expand with increasing Eu^2+^ doping concentration, whereas the volumes of [K(1)F_10_]^9−^ and [K(2)F_10_]^9−^ polyhedrons which are directly connected with [Eu(1)F_6_]^4−^ and [Eu(2)F_6_]^4−^ octahedrons both decrease, implying that the [K(1)F_10_]^9−^ and [K(2)F_10_]^9−^ polyhedrons are squeezed owing to the fact that the Ca(1)^2+^ and Ca(2)^2+^ sites are gradually occupied by the larger Eu^2+^ ions, which consequently results in a volume shrinkage of the [K(1)F_10_]^9−^ and [K(2)F_10_]^9−^ polyhedrons in the KCAF host lattice. These results evidently confirm the substitution of Ca^2+^ cations by the doped Eu^2+^ ions in the KCAF:Eu^2+^ phosphor. In addition, the schematic spatial views of the KCAF host and representative TB-type Sr_2_KNb_5_O_15_ compound are shown in Figure S2. By viewing along the [001] direction, the reticular framework of KCAF is analogical to that of Sr_2_KNb_5_O_15_ in which the octahedral [NbO_6_]^7−^ structure unit is equivalent to [AlF_6_]^3−^ octahedron in the crystal structure of KCAF [35]. As shown in Figure 2a,b, the structure framework of TB-type KCAF consists of the parallel [AlF_6_]^3−^_∞_ chains formed by the corner-sharing rigid [AlF_6_]^3−^ octahedrons, which further construct the highly cross–linked arrays stacked to each other.

Consequently, numerous tunnels surrounded by the [AlF_6_]^3−^ octahedrons along the [110] and [010] directions (denoted as the yellow dotted regions in Figure 2a,b) further provide the rigid crystallographic microenvironment in which two types of Ca^2+^ cations reside. Considering the common structural features of the aforementioned narrow-band UCr_4_C_4_-type silicate phosphors, it is concluded that the narrow emission bandwidth can not only be interpreted as the isotropic distribution of 5d orbitals of Eu^2+^ ions owing to the highly symmetrical dopant sites but can also be ascribed to the limitation of the rigid crystallographic microenvironment on the anisotropic structural relaxation of Eu^2+^ ions in their energetically different states [12,17]. As shown in Figure 2c, one can see that there are seven independent crystallographic sites in the crystal structure of the TB-type KCAF host lattice. K(1)^+^ and K(2)^+^ cations are coordinated by ten F^−^ anions to form [KF_10_]^9−^ polyhedrons. Ca(1)^2+^, Ca(2)^2+^, Al(1)^3+^, Al(2)^3+^, and Al(3)^3+^ cations are coordinated by six F^−^ anions to build [CaF_6_]^4−^ and [AlF_6_]^3−^ octahedrons, respectively. Since the [Ca(1)F_6_]^4−^ and [Ca(2)F_6_]^4−^ octahedrons are both asymmetric with different bond lengths, the rigid microenvironment of the Eu^2+^-occupied crystallographic Ca(1)^2+^ and Ca(2)^2+^ sites should be the primary determinant for its narrow-band emission. The rigid crystallographic microenvironment in which Eu^2+^ ions reside can effectively restrict the anisotropic local structural relaxation of Eu^2+^ ions in their energetically different states, which finally benefits by reducing the number of 5d excited states involved in the emission transition of KCAF:Eu^2+^ phosphor and thus realizes the narrow-band luminescence [36]. The rigid crystallographic microenvironment can also contribute to the suppression of the phonon energy associated with the thermally induced lattice vibration, resulting in a weak electron–phonon interaction even at a high temperature, which is essential for limiting the non-radiative transitions of Eu^2+^ ions in their high-energy 5d excited states [18]. Therefore, no additional non-radiative pathway is introduced into the down-conversion luminescence process with increasing temperature, which is conducive to realizing the desirable thermal stability of the TB-type KCAF:Eu^2+^ phosphor.

### 3.2. Morphology and Composition of KCAF:Eu^2+^

**Figure 3:**
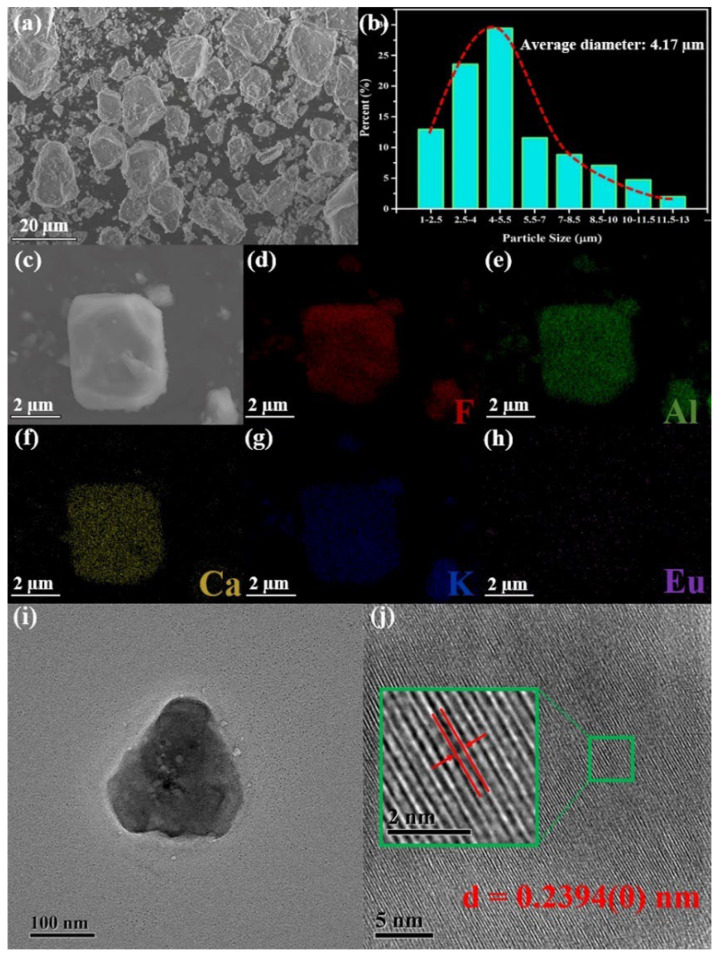
(**a**) SEM image of the representative KCAF:0.005Eu^2+^ phosphor. (**b**) Particle size distribution of KCAF:0.005Eu^2+^ phosphor. (**c**) SEM image of a selected KCAF:0.005Eu^2+^ phosphor particle. (**d**–**h**) EDS elemental mapping images of individual elements for the selected particle. (**i**) TEM and (**j**) high–resolution TEM images of KCAF:0.005Eu^2+^. The inset in (**j**) is the selected magnification area in the corresponding high–resolution TEM image.

It is widely believed that high crystallinity and uniform morphology can directly affect the luminescence performance of the phosphor. The SEM measurement of the representative TB-type KCAF:0.005Eu^2+^ phosphor was carried out, and the corresponding image and size distribution are illustrated in Figure 3a,b.

It is observed that the particles appear as irregular-shaped blocks with slight agglomeration owing to long-time, high-temperature sintering [10]. The size distribution of the particles is in the range of 1–13 µm with the average size of 4.17 µm. Furthermore, a typical particle of KCAF:0.005Eu^2+^ phosphor was selected for the elemental mapping analysis. As shown in Figure 3c–h, all the individual elements of F, Al, Ca, K, and Eu are homogeneously distributed over the whole particle without any trace amounts of element aggregation and phase separation, indicating the uniform chemical compositions of the studied KCAF:0.005Eu^2+^ phosphor. As shown in Figure S3, the EDS performed on the selected region of the particle indicates the measured atomic ratio of F:Al:Ca:K:Eu = 67.09:16.69:7.945:8.272:0.003, which is not entirely the same as the elementary proportion of KCAF:0.005Eu^2+^ phosphor yet approximates to its stoichiometric ratio. Figure 3i,j also gives the TEM and high-resolution TEM images of the typical microcrystal particle to verify the crystallinity of KCAF:0.005Eu^2+^ phosphor. The uniformly arranged lattice fringes with high resolution reveal the high crystalline stability of the representative TB-type KCAF:Eu^2+^ phosphor. The inter-planar spacing values of three different magnification areas shown in the inset of Figure 3j and Figure S4 are measured to be 0.2394(0), 0.2394(4), and 0.2395(1) nm, which are all convincingly indexed to the same (226) plane corresponding to the XRD peak position of 2θ = 37.535° in the refinement pattern; thereby, confirming the pure phase of our representative phosphor [28].

### 3.3. Valence State of Europium Ion

**Figure 4:**
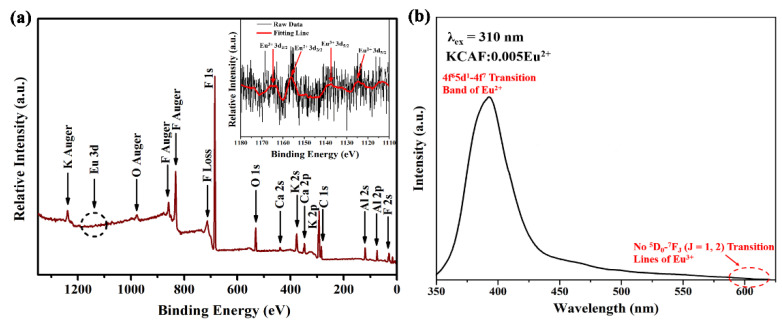
(**a**) XPS survey spectrum of KCAF:0.005Eu^2+^ phosphor. The inset is the high-resolution XPS spectrum for the Eu 3d positions of KCAF:0.005Eu^2+^ phosphor. (**b**) PL spectrum of KCAF:0.005Eu^2+^ phosphor (*λ*_ex_ = 310 nm).

Identifying the valence state of the doped europium ion can help to understand the PL properties of the TB-type KCAF:Eu^2+^ phosphor. The XPS survey spectrum of the KCAF:0.005Eu^2+^ phosphor is shown in Figure 4a.

It can be found that the photoelectron peaks of Al 2p, 2s, C 1s, K 2p, Ca 2p, K 2s, O 1s, and F 1s and auger peaks of F, O, and K appear at 74.8, 120.0, 284.2, 293.2, 347.9, 377.0, 531.1, 685.0, 833.0, 977.0, and 1238.0 eV, corresponding with the theoretical values of 72.9, 117.9, 285.0, 295.0, 346.6, 377.2, 531.8, 685.7, 833.0, 979.7, and 1235.0 eV, respectively [37]. The high-resolution XPS spectrum for the Eu 3d positions is shown in the inset of Figure 4a. Evidently, two characteristic peaks situated at 1155.1 and 1123.9 eV deriving from the 3d_3/2_ and 3d_5/2_ orbits of Eu^2+^ confirm the existence of the doped Eu^2+^ ion in the phosphor, which is responsible for its luminescence characteristic with the typical f–d transition as discussed below [38]. In addition, another two characteristic XPS peaks located at 1165.2 and 1137.6 eV and ascribed to the 3d_3/2_ and 3d_5/2_ orbits of the Eu^3+^ ion are also observed in the high-resolution XPS spectrum, indicating the existence of trace amounts of Eu^3+^ ion in our TB-type phosphor [39]. Such a mixed valence state of Eu in crystalline solids is mainly attributed to the interaction between the incomplete reduction of Eu^3+^ in the relatively weak reducing atmosphere of CO and the effect of surface oxidation [40]. Nevertheless, as exhibited in Figure 4b, the PL spectrum of our representative phosphor exhibits only one narrow emission band peaking at 400 nm owing to the characteristic 4f^6^5d^1^–4f^7^ transition of the Eu^2+^ ion, while no typical narrow ^5^D_0_–^7^F_J_ (J = 1, 2) transition lines of the Eu^3+^ ion are observed in the region of 590–620 nm. In addition, the PL spectrum of the sample excited by 395 nm is shown in Figure S5. It can be seen that the PL spectrum exhibits some narrow emission lines (λ_max_ = 591, 610 nm) originating from typical ^5^D_0_–^7^F_J_ (J = 1, 2) transitions of the Eu^3+^ ion. However, the emission intensity of the Eu^3+^ ion is very weak and even almost equal to the intensity of the baseline in the PL spectrum, which is much smaller than that of the Eu^2+^ ion at 310 nm excitation. Therefore, it is reasonably confirmed that the existence of the trace Eu^3+^ ion has no influence on the luminescence behavior of the Eu^2+^ ion in the TB-type KCAF:Eu^2+^ phosphor.

### 3.4. Electronic Band Structure

**Figure 5:**
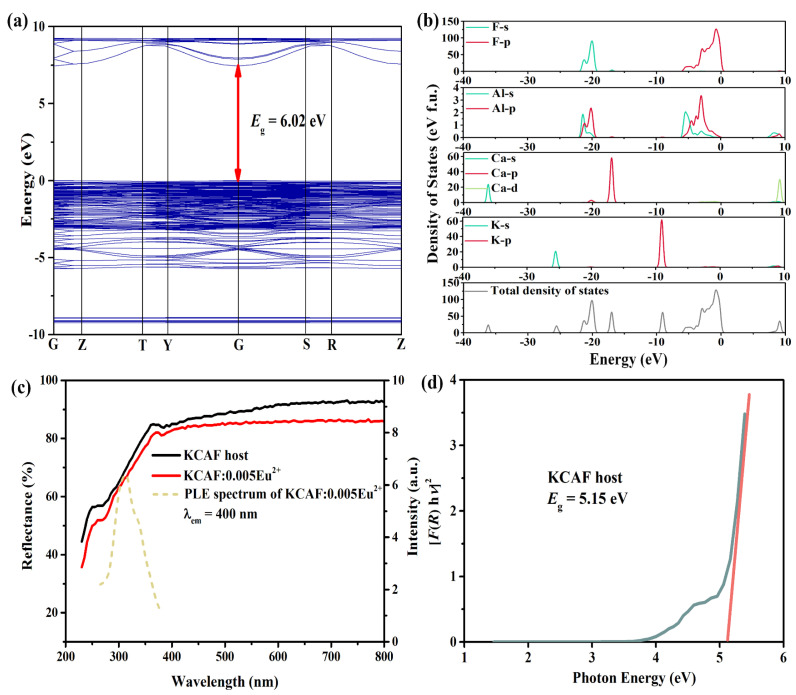
(**a**) Calculated electronic band structure and (**b**) total and partial densities of states for the KCAF host. (**c**) Diffuse reflection spectra of the KCAF host and KCAF:0.005Eu^2+^ phosphor as well as the PLE spectrum of KCAF:0.005Eu^2+^ phosphor monitored at 400 nm. (**d**) The relationship between [*F*(*R*)*hυ*]^2^ and *hυ* for the KCAF host.

The investigation of the electronic band structure of the host matrix can provide further insight into PL properties deriving from the electron transitions of the doped activator ions due to the significant role of the bandgap in setting positions of energy levels [41]. The band structure of the KCAF host obtained using the DFT calculations is presented in Figure 5a.

It is seen that both the energy maximum of the valence band and the energy minimum of the conduction band are located at point G, demonstrating the direct-gap feature of the TB-type KCAF host. It is generally accepted that phosphor with a large bandgap such as perovskite-type K_2_BaPO_4_F:Eu^2+^ (*E*_g_ = 6.16 eV, *I*_423K_/*I*_298K_ = 98%) and tridymite-type CaBe_2_(PO_4_)_2_:Eu^2+^ (*E*_g_ = 5.93 eV, *I*_423K_/*I*_298K_ = 105%) can efficiently prevent the thermally activated electrons in the 5d excited state from photoionizing into the conduction band, which is essential for maintaining their thermal stabilities [17,42]. The theoretical bandgap value of the TB-type KCAF host is calculated to be 6.02 eV, indicating that it cannot only accommodate both the ground and excited states of Eu^2+^ ions but also suppress the thermally ionized process that induces the excited 5d electrons into the conduction band at high temperature, thereby contributing to the thermally stable emission of KCAF:Eu^2+^ phosphor [43]. Moreover, the compositions of the electronic band structure of the KCAF host were investigated by the total and partial density of states (K, Ca, Al, and F atoms) as shown in Figure 5b. The low-energy region of the valence band stretching from −40 to −30 eV is dominated by the Ca-4s state, while the valence band in the energy region between −30 and −10 eV is contributed by K-4s, Ca-3p, Al-3s, Al-3p, and F-2s states. In addition, the energy region from −10 to 0 eV close to the Fermi level is primarily attributed to the contributions of K-3p, Al-3s, Al-3p, and F-2p states, and the bottom energy region of the conduction band is dominated by a Ca-4d state. In addition, it is observed that two obvious overlaps are located in the regions of −22–−19 and −6.5–0 eV owing to the hybridization of Al-3s, 3p, and F-2s states as well as Al-3s, 3p, and F-2p states, respectively, which suggest the formation of a [AlF_6_]^3−^ octahedron in the crystal structure of the TB-type KCAF host lattice. The [AlF_6_]^3−^ octahedrons are further involved in the construction of the structure framework of KCAF with the rigid microenvironment for dopant sites, which is essential for the thermally stable narrow-band emission [44,45].

The UV–visible diffuse reflection spectra of undoped and Eu^2+^-doped KCAF samples are shown in Figure 5c. One can see that the reflectance of the KCAF host in the region of 230–360 nm gradually increases owing to the host absorption [46]. Compared with the KCAF host, the diffuse reflection curve of KCAF:0.005Eu^2+^ phosphor shows a stronger absorption band in the same wavelength region, which demonstrates that the doping Eu^2+^ ions can effectively absorb the UV light, and thus are beneficial for realizing the down-conversion luminescence in the KCAF:Eu^2+^ phosphor [47]. The PLE spectrum of KCAF:0.005Eu^2+^ phosphor upon monitoring at 400 nm is also shown in Figure 5c for comparison. The excitation band ranging from 265 to 370 nm corresponds well with its characteristic absorption band in the diffuse reflection spectrum of KCAF:0.005Eu^2+^, further confirming that its enhanced absorption compared to the undoped KCAF host originates from the spin-allowed 4f^7^–4f^6^5d^1^ electric dipole transitions of Eu^2+^ ions [48]. Furthermore, the experimental bandgap value (*E*_g_) of the KCAF host is deduced by the extrapolation method as follows [49]:

(2) $${\left[F\left(R\right)h\upsilon \right]}^{2}\;=\;A\left(h\upsilon -E_{g}\right)$$

Here, *A* denotes the absorption constant and *hυ* represents the photon energy. *F*(*R*) is the absorptivity that can be calculated as follows [50]:

(3) $$F\left(R\right)\;=\;\frac{{\left(1-R\right)}^{2}}{2R}$$

Herein, *R* represents the absorption coefficient. Based on Equations (2) and (3), the experimental bandgap value of the KCAF host is calculated through the relationship between [*F*(*R*)*hυ*]^2^ and *hυ*. As shown in Figure 5d, by fitting the linear section of the curve and linearly extrapolating the value of [*F*(*R*)*hυ*]^2^ to zero, the experimental bandgap value of the KCAF host is estimated to be 5.15 eV, which is slightly smaller than the theoretical value of 6.018 eV calculated with DFT. This similar discrepancy also exists in other reported phosphors, such as Sr[Li_2_Al_2_O_2_N_2_]:Eu^2+^, as the experimental bandgap is smaller than the theoretical bandgap based on DFT [41].

### 3.5. PL Properties

**Figure 6:**
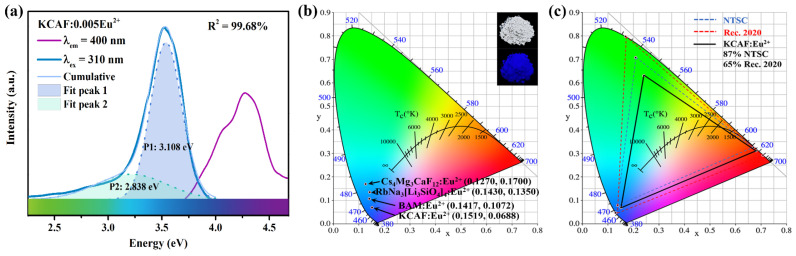
(**a**) PLE and PL spectra of KCAF:0.005Eu^2+^ phosphor (*λ*_em_ = 400 nm; *λ*_ex_ = 310 nm) and the Gaussian peaks fitting of the PL spectrum. (**b**) CIE chromaticity diagram for KCAF:Eu^2+^, BAM:Eu^2+^, RbNa_3_[Li_3_SiO_4_]_4_:Eu^2+^, and Cs_4_Mg_3_CaF_12_:Eu^2+^ phosphors under the excitation of 365 nm. The insets display the digital photographs of KCAF:Eu^2+^ phosphor under nature light and 365 lamp irradiation, respectively. (**c**) The color gamut of KCAF:Eu^2+^ phosphor as well as the NTSC and Rec. 2020 standards.

The PL and PLE spectra of the TB-type KCAF:0.005Eu^2+^ phosphor recorded at RT are depicted in Figure 6a.

Upon excitation at 310 nm, the PL spectrum exhibits an asymmetric emission band in its intense emission range of 2.75–3.54 eV (350–450 nm); perfectly overlapping the spectral cavity in the deep-blue region toward the sunlight-like full-spectrum lighting and aiming to improve the CRI in warm WLEDs. The small FWHM value (45 nm) of the TB-type KCAF:0.005Eu^2+^ phosphor is not only competitive with those of currently emerging, narrow-band, blue-emitting UCr_4_C_4_-type silicate phosphors [12,13,14,15] and deep-blue-emitting phosphors such as Ln_2_Si_2_O_7_:Bi^3+^ (Ln = Gd, Y, Lu, Sc; FWHM = 38–46 nm) [6] and Na_3_KMg_7_(PO_4_)_6_:Eu^2+^ (FWHM = 41 nm) [19] but also surpasses that of commercial BaMgAl_10_O_17_:Eu^2+^ (BAM:Eu^2+^, FWHM = 55 nm) [20] and other recently reported blue-emitting phosphors, for instance Cs_4_Mg_3_CaF_12_:Eu^2+^ (FWHM = 55 nm) [16], KLaSr_3_(PO_4_)_3_F:Eu^2+^ (FWHM = 53 nm) [21], and Rb_2_ZrSi_3_O_9_:Eu^2+^ (FWHM = 60 nm) [22] (Table 1). As discussed above, the rigid structure framework composed of numerous [AlF_6_]^3−^ octahedral units is conducive to the preferable narrow bandwidth feature of TB-type KCAF:Eu^2+^ phosphor owing to its restriction on the anisotropic structure relaxation of the Eu^2+^ ion in its energetically different states [51]. Furthermore, the asymmetric narrow emission band can be reasonably deconvoluted into two Gaussian components with the peaks at 3.108 (P1, 399 nm) and 2.838 eV (P2, 437 nm) in the energy scale of 2.25–3.59 eV (345–550 nm) [52]. This indicates the existence of two different luminescence centers (P1 for Eu(1)^2+^ luminescence center and P2 for Eu(2)^2+^ luminescence center) in the phosphor, which is consistent with the Rietveld refinement result of Eu^2+^ ions occupying the Ca(1)^2+^ and Ca(2)^2+^ sites. The emission peak position of phosphor is associated with the local crystal field environment of the Eu^2+^ ion. The larger crystal field splitting usually leads to the lower energy position of the 5d orbital accompanied by the smaller 4f^6^5d^1^–4f^7^ transition energy, resulting in its longer-wavelength emission [53]. The degrees of the crystal field splitting (*D*_q_) of Eu^2+^ ions located at different coordination environments are represented as follows [54]:

(4) $$D_{q}\;=\;\frac{{\mathrm{Ze}}^{2}r^{4}}{{6R}^{5}}$$

Here, *Z* and *e* represent the anion and electron charges, respectively, and *r* denotes the radius of the d wave function. *R* means the average distance from the central cation to its coordinated ions. It is seen that the splitting degree is inversely proportional to the value of *R*^5^, which suggests that the central cation with a shorter bond length would experience a larger crystal field splitting accompanied by a smaller energy of electron transition. Given the fact that the Eu(1)–F bond lengths are larger than those of Eu(2)–F in KCAF:Eu^2+^ phosphor, the splitting degree of the 5d energy level of the Eu^2+^ ion located at the Ca(1)^2+^ site is smaller than that of the Eu^2+^ ion in the Ca(2)^2+^ site, indicating that the P1 (peaking at 3.108 eV) and P2 (peaking at 2.838 eV) Gaussian components derive from the luminescence centers of Eu^2+^ ions in the Ca(1)^2+^ and Ca(2)^2+^ sites of the KCAF host lattice, respectively. The PLE spectrum of KCAF:0.005Eu^2+^ phosphor monitored at 400 nm shows a broad excitation band stretched from 3.26 (380 nm) to 4.68 eV (265 nm) with two characteristic peaks at 3.70 (335 nm) and 4 eV (310 nm), resulting from the typical 4f^7^–4f^6^5d^1^ transitions of Eu^2+^ ions [41]. In addition, Figure S6 depicts the PLE spectra of KCAF:0.005Eu^2+^ phosphor monitored at the different emission wavelengths of the P1 (3.108 eV, 399 nm) and P2 (2.838 eV, 437 nm) components. It is observed that the two PLE spectra show distinct shapes, finally evidencing the existence of two different luminescence centers in the TB-type KCAF:Eu^2+^ phosphor. Furthermore, as presented in Figure S7, the internal QE (IQE) and external QE (EQE) values of KCAF:0.01Eu^2+^ are determined to be 63% and 34%, respectively, which is superior to some recently reported blue phosphors, such as Sc_2_Si_2_O_7_:Bi^3+^ (EQE = 41%) [6], RbNa_3_[Li_3_SiO_4_]_4_:Eu^2+^ (IQE = 53%, EQE = 13%) [12], RbKLi_2_[Li_3_SiO_4_]_4_:Eu^2+^ (IQE = 50%) [14], and K_2_BaPO_4_F:Eu^2+^ (IQE = 50%, EQE = 25%) [17].

Color purity is one of the main performance indexes to evaluate the chromaticity characteristic of phosphor. The detailed Commission Internationale de l’Éclairage (CIE) chromaticity coordinates of the TB-type KCAF:Eu^2+^ phosphor calculated using the CIE 1931 color matching functions are illustrated in Figure 6b, and the chromaticity coordinates of the commercially available BAM:Eu^2+^ phosphor as well as the representative UCr_4_C_4_-type RbNa_3_[Li_3_SiO_4_]_4_:Eu^2+^ and perovskite-type Cs_4_Mg_3_CaF_12_:Eu^2+^ phosphors are given for comparison. The chromaticity coordinates of KCAF:Eu^2+^ are calculated to be (0.1519, 0.0688), which are closest to the standard blue point in comparison with the other three narrow-band blue-emitting phosphors. The inset of Figure 6b clearly exhibits the deep-blue luminescence of KCAF:Eu^2+^ phosphor under 365 nm lamp irradiation. The color purity of KCAF:Eu^2+^ phosphor is calculated as follows [55]:

(5) $$\mathrm{color}\;\mathrm{purity}\;=\;\frac{\sqrt{{\left({x-x}_{i}\right)}^{2}+{\left({y-y}_{i}\right)}^{2}}}{\sqrt{{\left(x_{d}-x_{i}\right)}^{2}+{\left(y_{d}-y_{i}\right)}^{2}}}\;\times \;100\%$$

Here, (*x*, *y*) represents the CIE chromaticity coordinates corresponding to the studied sample and (*x*_d_, *y*_d_) refers to the color coordinates corresponding to the dominant wavelength of the monochromatic light source. (*x*_i_, *y*_i_) is the chromaticity coordinates of the white illumination that equals (0.3333, 0.3333). According to Equation (5), the calculated color purity of KCAF:Eu^2+^ phosphor is 92%, exceeding those of RbNa_3_[Li_3_SiO_4_]_4_:Eu^2+^ (83%), Cs_4_Mg_3_CaF_12_:Eu^2+^ (85%), and BAM:Eu^2+^ (91%) phosphors, which exhibits the high color rendering performance of our phosphor [12,16,20]. The corresponding color gamut is further calculated based on the mixtures of deep-blue-emitting KCAF:Eu^2+^ and commercial green/red-emitting β-SiAlON:Eu^2+^/K_2_SiF_6_:Mn^4+^ phosphors for evaluating the application potential of our TB-type KCAF:Eu^2+^ phosphor. As shown in Figure 6c, the calculated color gamut can cover 87% of the NTSC standard and 65% of the Rec. 2020 standard in CIE 1931 color space, which is larger than those of other aforementioned blue phosphors, viz., RbNa_3_[Li_3_SiO_4_]_4_:Eu^2+^ (75% of NTSC) [12], BAM:Eu^2+^ (82% of NTSC) [20], and Sr_3_Lu_2_Ge_3_O_12_:Bi^3+^ phosphors (83% of NTSC) [56].

**Figure 7:**
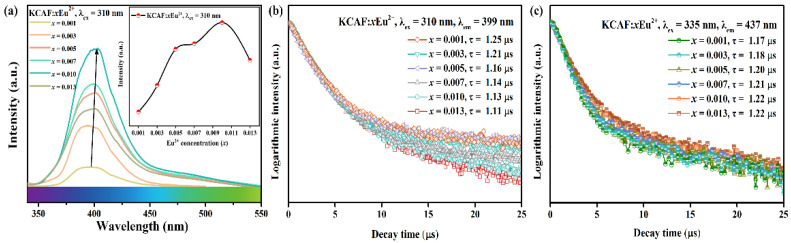
(**a**) PL spectra of KCAF:*x*Eu^2+^ (*x* = 0.001–0.013) phosphors (λ_ex_ = 310 nm). The inset shows the variation of emission intensities of KCAF:*x*Eu^2+^ (*x* = 0.001–0.013) phosphors versus Eu^2+^ doping concentration. (**b**) The fluorescence decay curves of KCAF:*x*Eu^2+^ (*x* = 0.001–0.013) phosphors with 310 nm excitation and 399 nm emission. (**c**) The fluorescence decay curves of KCAF:*x*Eu^2+^ (*x* = 0.001–0.013) phosphors with 335 nm excitation and 437 nm emission.

The concentration-dependent PL spectra of TB-type KCAF:*x*Eu^2+^ (*x* = 0.001–0.013) phosphors excited at 310 nm are further shown in Figure 7a. 

Apparently, all the PL spectra show a similar narrow-band emission around 400 nm with FWHM close to 45 nm, resulting from the 4f^6^5d^1^–4f^7^ emission transition of Eu^2+^ ions [16]. As shown in the inset of Figure 7a, the emission intensity of KCAF:*x*Eu^2+^ gradually increases with the increase in Eu^2+^ doping content and reaches its maximum at *x* = 0.01; then, the intensity decreases on account of the concentration quenching effect [16]. The mechanism of concentration quenching of KCAF:Eu^2+^ phosphor is explained as the non-radiative energy transfer (ET) between the neighboring Eu^2+^ ions caused by the exchange or electric multipolar interactions, which can be reasonably determined by the critical distance (*R*_c_) between Eu^2+^ ions as follows [57]:

(6) $$R_{c}\approx 2{\left(\frac{3V}{{4\pi X}_{c}Z}\right)}^{1/3}$$

in which *V* corresponds to the cell volume of the KCAF host (*V* = 1994.750(4) Å^3^), *X*_c_ means the critical concentration of Eu^2+^ ions (*X*_c_ = 0.01), and *Z* is the number of host cations per unit cell (*Z* = 12). In general, the non-radiative ET is dominated by exchange interaction only when the distance between the adjacent activators is within 5 Å [58]. Based on Equation (6), *R*_c_ is calculated with the value of 31.66 Å which is larger than 5 Å, demonstrating that the electric multipolar interaction is mainly responsible for the non-radiative ET process in the Eu^2+^-doped KCAF phosphor. Based on Dexter’s theory, the detailed mechanism of electric multipolar interaction is further verified as follows [59]:

(7) $$\frac{I}{x}\;=\;\frac{K}{{1\;+\;\beta (x)}^{\theta /3}}$$

Herein, *I*/*x* represents the emission intensity (*I*) per doping concentration (*x*) for Eu^2+^ ions, and *K* and *β* mean the constants for the KCAF host under the same excitation conditions. *θ* is the index that determines the type of electric multipolar interaction, where *θ* = 6, 8, and 10 represent the dipole–dipole, dipole–quadrupole, and quadrupole–quadrupole interactions, respectively [60]. As depicted in Figure S8 in the ESM, the dependence of $\mathrm{lg}(I/x_{{\mathrm{Eu}}^{2+}})$ on ${\mathrm{lg}(x}_{{\mathrm{Eu}}^{2+}})$ is determined to be a linear relation with a slope value of −1.898. Therefore, the value of *θ* is approximately 6, inferring the dominance of the dipole–dipole interaction in the concentration quenching of Eu^2+^ ions in the TB-type KCAF:Eu^2+^ phosphor. Figure S9a in the ESM depicts the fluorescence decay curves of KCAF:*x*Eu^2+^ monitored at the emission wavelength of 400 nm under 310 nm excitation. The average lifetime (*τ*) can be calculated via Equation (8) [61]:

(8) $$\tau \;=\;\frac{\int_{0}^{\infty }\mathrm{tI}(t)\mathrm{dt}}{I(t)\mathrm{dt}}$$

therefore, the decay lifetimes for KCAF:*x*Eu^2+^ (*x* = 0.001–0.013) phosphors are calculated to be 1.27, 1.24, 1.22, 1.18, 1.17, and 1.13 µs, respectively. One can see that the average lifetime gradually decreases from 1.27 to 1.13 µs with Eu^2+^ content increasing from 0.001 to 0.013, which can be interpreted as enhancing the non-radiative ET between Eu^2+^ ions with increasing Eu^2+^ doping content [55]. The ion–ion non-radiative ET could be further interpreted by the Inokuti–Hirayama model [62]:

(9) $$I(t)=I_{0}\exp[-\frac{t}{\tau_{0}}-Qt^{3/S}]$$

Here, *I*(*t*) and *I*_0_ mean the emission intensities at time *t* and 0. *τ*_0_ means the intrinsic lifetime of the donors. S = 6, 8, and 10 represent the dipole–dipole, dipole–quadrupole, and quadrupole–quadrupole interactions, respectively. *Q* is the energy transfer parameter, which is described as follows:

(10) $$Q=\frac{4\pi }{3}C_{A}\Gamma (1-\frac{3}{S}){\left[C_{\mathrm{DA}}^{\left(S\right)}\right]}^{3/S}$$

where *C_A_* and *C^(S)^_DA_* refer the concentrations of acceptors and the ET microparameter. Γ is the gamma function. The time-dependent luminescence intensity of KCAF:0.005Eu^2+^ ion was fitted to the above equations until a good fit was achieved, as shown in Figure S9b. It can be seen that the best fit between the decay curve and the Inokuti–Hirayama model is obtained for *S* = 6 with the fitting degree *R*^2^ = 0.99813, which indicates that the mechanism for ET among Eu^2+^ ions in KCAF:Eu^2+^ phosphor is mainly driven by a dipole–dipole interaction. This result is consistent with that of PL spectra, further confirming the dipole–dipole interaction among Eu^2+^ ions. In addition, as shown in Figure S10, a significant spectral overlap in the range of 345–390 nm is observed between the PL spectrum of Eu(1)^2+^ luminescence center excited at 310 nm and PLE spectrum of Eu(2)^2+^ luminescence center monitored at 437 nm, indicating the possibility of ET from Eu(1)^2+^ to Eu(2)^2+^ luminescence centers in the TB-type KCAF:Eu^2+^ phosphor. Based on the Dexter’s theory of the dipole–dipole interaction, the value of *R*_c_ can also be obtained by the experimental spectral data of the overlapped PLE and PL spectra as follows [46]:

(11) $$R_{c}^{6}\;=\;0{.63\;\times \;10}^{28}\frac{4{.8\;\times \;10}^{-16}P_{A}}{E^{4}}\int F_{s}\left(E\right)F_{A}\left(E\right)dE$$

Herein, *P*_A_ means the oscillator strength of the electric dipole transition for the energy-accepting ion (0.02 for Eu^2+^), and *E* stands for the maximum energy of spectral overlap. ∫*F*_s_(*E*)*F*_A_(*E*)d*E* means the spectral overlap integral between the normalized PLE and PL spectra of KCAF:Eu^2+^ phosphor. The energy of *E* and the spectral overlap ∫*F*_s_(*E*)*F*_A_(*E*)d*E* are calculated with the values of 2.19 eV and 0.2486 eV^−1^, respectively. Based on the above parameters, the *R*_c_ value is calculated to be 29.46 Å, which matches the result of 31.66 Å calculated by Equation (6). For further verification of the ET process from Eu(1)^2+^ to Eu(2)^2+^ luminescence centers, the fluorescence decay curves of Eu(1)^2+^ (*λ*_ex_ = 310 nm, *λ*_em_ = 399 nm), and Eu(2)^2+^ (*λ*_ex_ = 335 nm, *λ*_em_ = 437 nm) luminescence centers in KCAF:*x*Eu^2+^ phosphors are shown in Figure 7b,c. According to Equation (8), the decay lifetimes for the Eu(1)^2+^ luminescence center are calculated to be 1.25, 1.21, 1.16, 1.14, 1.13, and 1.11 µs, while those for the Eu(2)^2+^ luminescence center are 1.17, 1.18, 1.20, 1.21, 1.22, and 1.22 µs. One can see that the lifetime of the Eu(2)^2+^ luminescence center increases from 1.17 µs at *x* = 0.001 to 1.22 µs at *x* = 0.013 rather than gradually decreasing as that of the Eu(1)^2+^ luminescence center. This is attributed to the ET from Eu(1)^2+^ to Eu(2)^2+^ luminescence centers, efficiently competing with the spontaneous non-radiative transitions of Eu(2)^2+^ luminescence center in its high-energy 5d excited states. This distinct variation in the decay lifetimes of Eu(1)^2+^ and Eu(2)^2+^ luminescence centers evidently verifies the ET process from Eu(1)^2+^ to Eu(2)^2+^ in the TB-type KCAF:Eu^2+^ phosphor.

### 3.6. Thermal Stability

**Figure 8:**
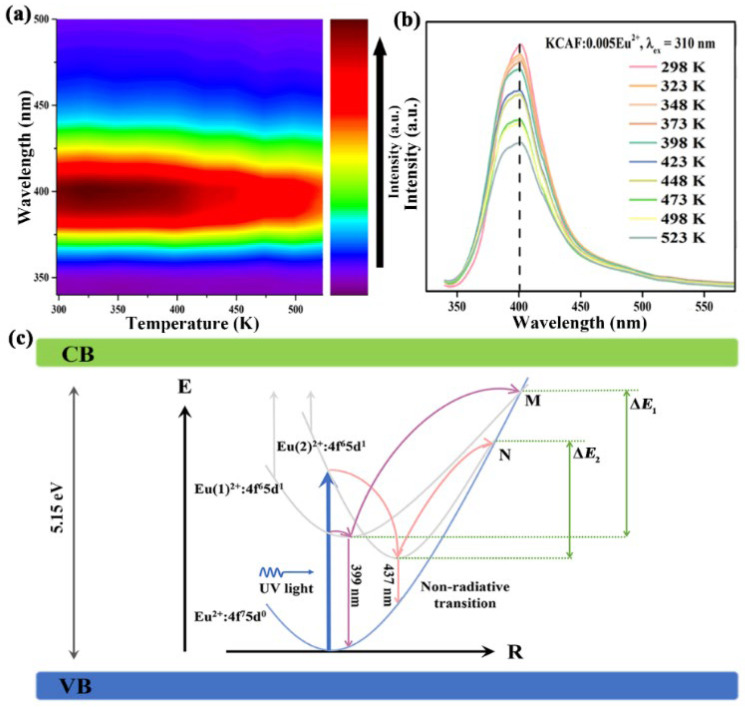
(**a**) Contour plot of the PL spectra of KCAF:0.005Eu^2+^ phosphor as a function of temperature under 310 nm excitation. (**b**) Temperature-dependent PL spectra of KCAF:0.005Eu^2+^ phosphor under 310 nm excitation. (**c**) Schematic illustration of the configurational coordinate diagram of the 4f^7^5d^0^ ground state and 4f^6^5d^1^ excited state for Eu^2+^ ions in the TB-type KCAF:Eu^2+^ phosphor.

Thermal stability has always been a concerned technological parameter of phosphor for WLED applications because of its significant influence on the light output and service life of the fabricated devices at high operating temperatures of T ≥ 423 K [63]. The temperature-dependent PL spectra of the TB-type KCAF:0.005Eu^2+^ phosphor are shown in Figure 8a,b.

With an increase in the temperature from 298 to 523 K, a series of PL spectra still retain the narrow-band spectral profile with a stable peak position at 400 nm and a slight broadening of the bandwidth from 45 to 50 nm. The behavior of thermally induced emission band broadening in the KCAF:0.005Eu^2+^ phosphor can be explained by the following equation [64]:

(12) $$\mathrm{FWHM}\left(T\right)\;=\;h\upsilon {\left[8\;\times \;\ln2\;\times \;S\;\times \;\coth(\frac{h\upsilon }{2\mathrm{kT}})\right]}^{1/2}$$

Herein, *S* is the Huang–Rhys parameter, *hυ* represents the vibrational phonon energy, *k* is the Boltzmann constant (8.62 × 10^−5^ eV·K^−1^), and *T* means the certain temperature. According to Equation (12), the emission bandwidth is positively related to the temperature *T*, indicating that an increase in temperature can cause a broadening behavior of the emission band. However, the broadening emission bandwidth of KCAF:0.005Eu^2+^ phosphor is only limited within 5 nm even at 523 K owing to the restriction of a non-radiative transition of Eu^2+^ ions resulting from the suppression of thermally enhanced lattice vibration by the rigid network of the KCAF host, which is beneficial to lighting and display devices for maintaining their color saturation at high operating temperature [65]. Furthermore, a slight decrease in the emission intensity is observed due to the thermal quenching effect that can be interpreted as the intensification of the non-radiative transition of Eu^2+^ ion in its excited state [44]. The emission intensity of KCAF:0.005Eu^2+^ phosphor drops only 10% at 423 K compared to that at RT, which is not only superior to some recently reported blue phosphors such as Cs_4_Mg_3_CaF_12_:Eu^2+^ (*I*_423K_/*I*_298K_ = 82%) [16], RbKLi_2_[Li_3_SiO_4_]_4_:Eu^2+^ (*I*_423K_/*I*_298K_ = 88%) [14], and Rb_2_ZrSi_3_O_9_:Eu^2+^ (*I*_423K_/*I*_298K_ = 82%) [22], but also competitive with the commercial blue phosphor BAM:Eu^2+^ (*I*_373K_/*I*_298K_ = 91%) [20] (Table 1). The configurational coordinate diagram is utilized to elucidate the spin-allowed f–d transitions of Eu^2+^ ions in order to understand the thermal quenching behavior of KCAF:Eu^2+^ phosphor, as illustrated in Figure 8c. Pumped with UV light, the electrons of the two luminescence centers transfer from the 4f^7^5d^0^ ground state to the 4f^6^5d^1^ excited state, and they further relax to the lowest excited state through a non-radiative transition. Finally, the narrow-band blue luminescence composed of two components peaking at 399 and 437 nm are realized based on the electric-dipole allowed 4f^6^5d^1^–4f^7^5d^0^ transition of Eu^2+^ ions [42]. Nevertheless, with an increase in temperature, the thermally activated electrons can overcome their energy barriers (Δ*E*_1_ and Δ*E*_2_) with the assistance of enhanced electron–phonon interactions to reach the crossing points between their respective excited state and ground state (M and N) and then transfer to the ground state through non-radiative transition resulting in the emission loss at a higher temperature. Whereas, the non-radiative transition involved in the thermal quenching process can be significantly restricted owing to the intrinsic structure feature of KCAF:Eu^2+^ phosphor; for example, the rigid TB-type structure framework composed of numerous [AlF_6_]^3−^ octahedrons can largely suppress the electron–phonon interaction. In addition, its large bandgap characteristic also endows KCAF:Eu^2+^ phosphor with the ability to prevent the thermally activated excited electrons from being ionized into the conduction band, thus enabling the excited electrons to return to the ground state through the radiative transition which is beneficial for the thermal stability of KCAF:Eu^2+^ phosphor.

**Figure 9:**
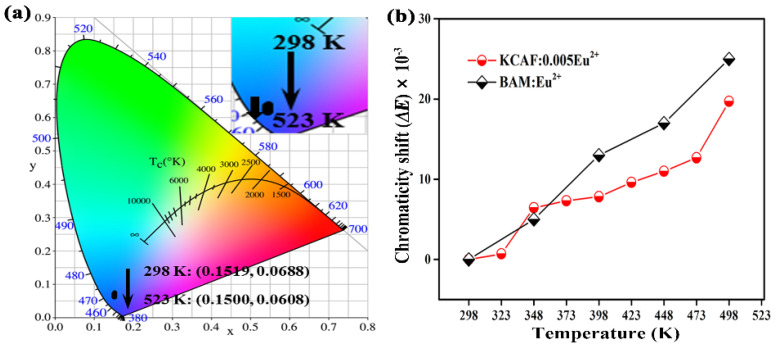
(**a**) Coordinate shift of CIE chromaticity diagram for KCAF:0.005Eu^2+^ phosphor with temperature increasing from 298 to 523 K. (**b**) Chromaticity shifts of KCAF:0.005Eu^2+^ and BAM:Eu^2+^ phosphors as a function of temperature.

To further reveal the temperature dependence of emission intensity associated with the electron–phonon interaction, the thermal quenching activation energy (Δ*E*) for KCAF:Eu^2+^ phosphor is calculated through the Arrhenius equation [55]:

(13) $$I_{T}=\frac{I_{0}}{1+c\exp(-\frac{\Delta E}{kT})}$$

in which *I*_T_ and *I*_0_ are the emission intensities at a given temperature *T* and RT. *c* is a constant of the given host and *k* denotes the Boltzmann constant (8.62 × 10^−5^ eV·K^−1^). As depicted in Figure S11, the relationship between ln[(*I*_0_/*I*_T_) − 1] and 1/*kT* is fitted to be linear with a slope of −0.23, which indicates that the value of Δ*E* for KCAF:0.005Eu^2+^ phosphor is 0.23 eV. Theoretically, the activation energy Δ*E* can be interpreted as the energy barrier for the thermally activated excited electrons to reach a high-energy 5d excited state located at the crossing point of the 4f ground state and the 5d excited state. Thus, a larger value of Δ*E* indicates a greater difficulty for thermally activated excited electrons to reach the high-energy 5d excited state, which reduces the non-radiative transition of Eu^2+^ ion in its excited state and assists KCAF:Eu^2+^ phosphor with the thermally stable emission. The comparison of comprehensive PL performances of TB-type KCAF:Eu^2+^ and other recently emerging blue or deep-blue phosphors shown in Table 1 demonstrates that our TB-type KCAF:Eu^2+^ phosphor (color purity = 92%, color gamut = 87% of NTSC, 65% of Rec. 2020, and *I*_423K_/*I*_298K_ = 90%) exhibits both the larger color gamut and superior thermal stability in comparison with some of the listed phosphors such as RbNa_3_[Li_3_SiO_4_]_4_:Eu^2+^ (color purity = 83%, color gamut = 75% of NTSC, 56% of Rec. 2020) [12], K_2_BaPO_4_F:Eu^2+^ (color gamut = 83% of NTSC, 62% of Rec. 2020) [17], Cs_4_Mg_3_CaF_12_:Eu^2+^ (color purity = 85%, *I*_423K_/*I*_298K_ = 82%) [16], and Rb_2_ZrSi_3_O_9_:Eu^2+^ (*I*_423K_/*I*_298K_ = 82%) [22], thereby demonstrating its potential application in full-spectrum warm WLED devices. To assess the chromaticity stability of narrow-band deep-blue-emitting KCAF:Eu^2+^ phosphor, the temperature-dependent chromaticity coordinates of KCAF:0.005Eu^2+^ phosphor are also calculated and the corresponding CIE chromaticity points are plotted in the CIE chromaticity diagram (Figure 9a).

As the temperature increases from RT to 523 K, the deep-blue emission color of KCAF:0.005Eu^2+^ phosphor shows a slight blue shift with chromaticity coordinates varying from (0.1519, 0.0688) to (0.1500, 0.0608). To evaluate the color consistency of KCAF:Eu^2+^ phosphor, the chromaticity shift (Δ*E*) is estimated as follows [66]:

(14) $${\Delta E\;=\;[(u}_{t}{\;-\;u}_{0}{)}^{2}{+(v}_{t}\;-{\;v}_{0}{)}^{2}{+(w}_{t}\;-{\;w}_{0}{)}^{2}{]}^{1/2}$$

Herein, *u* = 4*x*/(3 − 2*x* + 12*y*), *v* = 9*y*/(3 − 2*x* + 12*y*), and *w* is determined as 1 − *u* − *v*. (*u*, *v*) and (*x*, *y*) represent the chromaticity coordinates in the *uv* uniform color space and the chromaticity coordinates of phosphor in CIE 1931 color space, respectively. *t* and 0 correspond to the given temperature and initial temperature (RT), respectively. According to Equation (14), the temperature-dependent chromaticity shifts of KCAF:0.005Eu^2+^ and commercial BAM:Eu^2+^ phosphors are shown in Figure 9b. It is observed that the chromaticity shifts of the two phosphors gradually increase with the increasing temperature, in which the chromaticity shift of BAM:Eu^2+^ is determined to be 20.20 × 10^−3^ at 473 K, while KCAF:0.005Eu^2+^ displays a smaller chromaticity shift of 12.67 × 10^−3^ at 473 K, indicating the high color consistency of our TB-type KCAF:Eu^2+^ phosphor for practical application in bridging the deep-blue spectral cavity toward sunlight-like full-spectrum lighting.

## 4. Conclusions

Inspired by the unique structural feature of TB-type minerals, a novel narrow-band deep-blue-emitting KCAF:Eu^2+^ phosphor was developed for the first time. Upon excitation with UV light, the PL spectrum of KCAF:Eu^2+^ shows the novel deep-blue emission band centered at 400 nm with a narrow FWHM of 45 nm originating from the rigid structure framework of the KCAF host, which can limit the anisotropic structure relaxation of Eu^2+^ in its energetically different states and thus exhibit a narrow emission bandwidth. Owing to the restriction of a non-radiative transition of Eu^2+^ ions resulting from the suppression of the thermally enhanced lattice vibration by its rigid structure framework, the KCAF:Eu^2+^ phosphor further exhibits favorable thermal stability with low emission loss (*I*_423K_/*I*_298K_ = 90%) and a slight chromaticity shift (12.67 × 10^−3^ at 473 K). Our developed KCAF:Eu^2+^ phosphor also possesses a high color purity up to 92% and a large color gamut covering 87% of the NTSC standard, which is superior to other currently emerging narrow-band deep-blue-emitting phosphors. Our present work not only demonstrates the great application potential of narrow-band deep-blue-emitting TB-type KCAF:Eu^2+^ phosphor as a promising candidate for applications in bridging the deep-blue spectral cavity toward sunlight-like full-spectrum lighting, but also provides a direction for developing novel narrow-band phosphor, based on the natural mineral structure.

## Figures and Tables

**Table 1:** Luminescence characteristics of TB-type KCAF:Eu^2+^ phosphor and some representative narrow-band blue- or deep-blue-emitting phosphors.

Phosphors	Peak (nm)	FWHM (nm)	Thermal Stability	Color Purity (%)	Color Gamut (%NTSC)	Color Gamut (%Rec. 2020)	IQE (%)	EQE (%)
Gd_2_Si_2_O_7_:Bi^3+^ [6]	422 (deep blue)	46	58% (423 K)	–	–	–	–	–
Y_2_Si_2_O_7_:Bi^3+^ [6]	412 (deep blue)	41	70% (423 K)	–	–	–	–	–
Lu_2_Si_2_O_7_:Bi^3+^ [6]	408 (deep blue)	39	53% (423 K)	–	–	–	–	–
Sc_2_Si_2_O_7_:Bi^3+^ [6]	403 (deep blue)	38	81% (423 K)	–	–	–	41	–
RbNa_3_[Li_3_SiO_4_]_4_:Eu^2+^ [12]	471 (blue)	22	96% (423 K)	83	75	56	53	13
RbNa_2_K[Li_3_SiO_4_]_4_:Eu^2+^ [13]	480 (blue)	26	99% (423 K)	–	–	–	–	–
RbKLi_2_[Li_3_SiO_4_]_4_:Eu^2+^ [14] Na_0.5_K_0.5_[Li_3_SiO_4_]:Eu^2+^ [15]	474 (blue) 486 (blue)	25 21	88% (423 K) 93% (423 K)	– 67	– –	– –	50 76	– 30
Cs_4_Mg_3_CaF_12_:Eu^2+^ [16]	474 (blue)	55	82% (423 K)	85	–	–	64	–
K_2_BaPO_4_F:Eu^2+^ [17]	439 (blue)	25	98% (423 K)	99	83	62	50	25
BaAl_12_O_19_:Eu^2+^ [18]	443 (blue)	52	92% (473 K)	90	90	67	90	–
Na_3_KMg_7_(PO_4_)_6_:Eu^2+^ [19]	446 (blue)	41	97% (497 K)	95	–	–	93	40
BAM:Eu^2+^ [20]	453 (blue)	55	91% (373 K)	91	82	61	89	–
KLaSr_3_(PO_4_)_3_F:Eu^2+^ [21] Rb_2_ZrSi_3_O_9_:Eu^2+^ [22]	461 (blue) 470 (blue)	53 60	51% (473 K) 82% (423 K)	– –	79 –	59 –	65 75	– –
KCAF:Eu^2+^	400 (deep blue)	45	90% (423 K)	92	87	65	63	34

**Table 2:** Crystallographic parameters of the KCAF host, KCAF:0.005Eu^2+^, and KCAF:0.013Eu^2+^ phosphors obtained from XRD Rietveld refinements.

Formula	KCAF	KCAF:0.005Eu^2+^	KCAF:0.013Eu^2+^
Crystal system	Orthorhombic	Orthorhombic	Orthorhombic
Profile range (^o^)	5–130	5–130	5–130
Radiation type; λ (Å)	X-ray; 1.5418	X-ray; 1.5418	X-ray; 1.5418
Temperature (K)	298	298	298
Space group (*Z*)	*C*222_1_ (20); (12)	*C*222_1_ (20); (12)	*C*222_1_ (20); (12)
*a* (Å)	12.329 (6)	12.329 (8)	12.331 (7)
*b* (Å)	7.152 (7)	7.154 (6)	7.156 (3)
*c* (Å)	22.618 (8)	22.619 (3)	22.623 (8)
Unit cell volume *V* (Å^3^)	1994.750 (4)	1995.356 (7)	1996.535 (5)
Weighted profile *R*-factor, *R*_wp_ (%)	6.32	6.57	6.78
Profile *R*-factor, *R*_p_ (%)	4.36	4.25	4.37
*χ* ^2^	2.14	2.43	2.58

## Data Availability

Data are available upon request due to restrictions, e.g., privacy or ethical.

## Supplementary Materials

The following supporting information can be downloaded at: https://www.mdpi.com/article/10.3390/ma16145053/s1, Table S1: The refinement results of the atomic positions and site occupancies of TB-type KCAF:0.005Eu^2+^ and KCAF:0.013Eu^2+^ phosphors; Figure S1: (**a**) The coordination environments of K^+^ and Eu^2+^ cations in the KCAF host lattice. (**b**) Changes in the volume for unit cell, [K(1)F_10_]^9−^, and [K(2)F_10_]^9−^ polyhedrons; Figure S2: Crystal structure of (**a**) KCAF and (**b**) Sr_2_KNb_5_O_15_ hosts from the [001] direction; Figure S3: Energy-dispersive spectrum of KCAF:0.005Eu^2+^ phosphor. The inset is the respective atom percentage of KCAF:0.005Eu^2+^; Figure S4: High-resolution TEM image of KCAF:0.005Eu^2+^. The insets are the selected magnification areas in the corresponding high-resolution TEM image; Figure S5: PL spectrum of KCAF:0.005Eu^2+^ phosphor (λ_ex_ = 395 nm); Figure S6: PLE spectra of KCAF:0.005Eu^2+^ phosphor monitored at 399 and 437 nm; Figure S7: Excitation line of BaSO_4_ and emission spectrum of the KCAF:0.01Eu^2+^ phosphor. The inset is the magnification of the emission spectrum; Figure S8: Line-fitting relationship of $\mathrm{lg}(I/x_{{\mathrm{Eu}}^{2+}})$ versus ${\mathrm{lg}(x}_{{\mathrm{Eu}}^{2+}})$; Figure S9: (**a**) The fluorescence decay curves of KCAF:*x*Eu^2+^ (*x* = 0.001–0.013) phosphors with 310 nm excitation and 400 nm emission. (**b**) The fluorescence decay curves of KCAF:0.005Eu^2+^ phosphor. The solid lines correspond to the fits to the Inokuti–Hirayama model for S = 6, 8, and 10; Figure S10: The PLE and PL spectra of KCAF:0.005Eu^2+^ phosphor (*λ*_em_ = 437 nm; *λ*_ex_ = 310 nm); Figure S11: The activation energy for thermal quenching of KCAF:0.005Eu^2+^ phosphor.
