# Supplementary material for: Narrow-Band Deep-Blue Emission and Superior Thermal Stability of Fluoroaluminate Phosphor Based on Tungsten Bronze-Type Mineral Structure
Source: Materials (Basel). 2023 Jul 17;16(14):5053. doi: 10.3390/ma16145053 (PMC10383773; doi:10.3390/ma16145053)
Supplement: Supplementary file 1 [file materials-16-05053-s001.zip › materials-2481833-supplementary.pdf]

materials

Supplementary Materials

Narrow-Band Deep-Blue Emission and Superior Thermal Stability of Fluoroaluminate Phosphor based on Tungsten Bronze-Type Mineral Structure

Rui Lu and Jianfeng Sun *

[1] School of Materials Science and Engineering, Hebei University of Technology, Tianjin, 300130, China; m13230321575@163.com
[2] Key Laboratory of Special Functional Materials for Ecological Environment and Information (Hebei University of Technology), Ministry of Education, Tianjin 300130, China
* Correspondence: sunjianfeng10@hebut.edu.cn

Table S1. The refinement results of the atomic positions and site occupancies of TB-type KCAF:0.005Eu^{2+} and KCAF:0.013Eu^{2+} phosphors.

KCAF:0.005Eu^{2+}					KCAF:0.013Eu^{2+}				
Atom	x	y	z	occupancy	Atom	x	y	z	occupancy
K1	0.0094	0.0000	0.0000	1.0000	K1	0.0094	0.0000	0.0000	1.0000
K2	−0.0218	0.0143	0.1667	1.0000	K2	−0.0218	0.0143	0.1667	1.0000
Ca1	0.2443	0.0258	0.0834	0.9970	Ca1	0.2443	0.0258	0.0834	0.9910
Ca2	0.0000	0.5201	0.2500	0.9980	Ca2	0.0000	0.5201	0.2500	0.9960
Al1	−0.0080	0.4991	0.0799	1.0000	Al1	−0.0080	0.4991	0.0799	1.0000
Al2	0.2398	0.2428	−0.0864	1.0000	Al2	0.2398	0.2428	−0.0864	1.0000
Al3	0.2481	0.7696	0.2468	1.0000	Al3	0.2481	0.7696	0.2468	1.0000
F1	0.2092	0.7848	0.0106	1.0000	F1	0.2092	0.7848	0.0106	1.0000
F2	−0.0289	0.5124	0.1558	1.0000	F2	−0.0289	0.5124	0.1558	1.0000
F3	0.5132	0.0000	0.0000	1.0000	F3	0.5132	0.0000	0.0000	1.0000
F4	0.2713	0.7326	0.1669	1.0000	F4	0.2713	0.7326	0.1669	1.0000
F5	0.2751	0.3134	0.1773	1.0000	F5	0.2751	0.3134	0.1773	1.0000
F6	0.0539	0.2796	0.0841	1.0000	F6	0.0539	0.2796	0.0841	1.0000
F7	−0.0675	0.2725	−0.0702	1.0000	F7	−0.0675	0.2725	−0.0702	1.0000
F8	0.8902	0.2464	0.2574	1.0000	F8	0.8902	0.2464	0.2574	1.0000
F9	0.8914	0.7792	0.2681	1.0000	F9	0.8914	0.7792	0.2681	1.0000
F10	0.1262	0.6017	0.0939	1.0000	F10	0.1262	0.6017	0.0939	1.0000
F11	0.1421	0.4030	0.4284	1.0000	F11	0.1421	0.4030	0.4284	1.0000
F12	0.1660	−0.0402	0.1034	1.0000	F12	0.1660	−0.0402	0.1034	1.0000
F13	0.1805	0.0540	0.4269	1.0000	F13	0.1805	0.0540	0.4269	1.0000
F14	0.2318	0.5180	0.2617	1.0000	F14	0.2318	0.5180	0.2617	1.0000
Eu1	0.2443	0.0258	0.0834	0.0030	Eu1	0.2443	0.0258	0.0834	0.0090
Eu2	0.0000	0.5201	0.2500	0.0020	Eu2	0.0000	0.5201	0.2500	0.0040

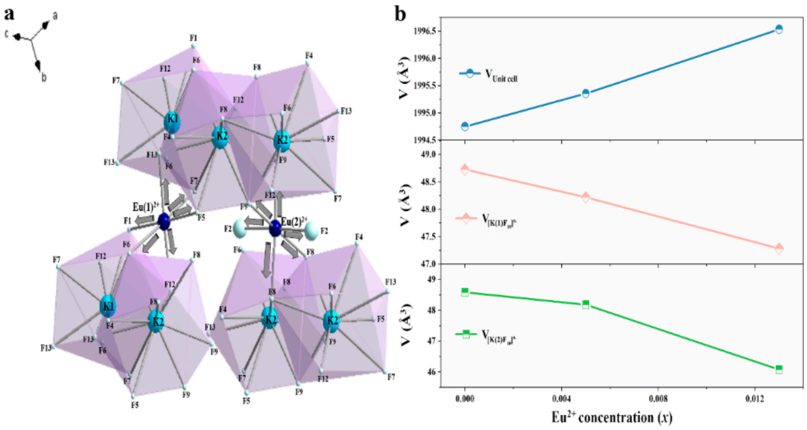

Figure S1. (a) The coordination environments of K^+ and Eu^{2+} cations in the KCAF host lattice. **(b)** Changes of the volume for unit cell, $[K(1)F_{10}]^{9-}$, and $[K(2)F_{10}]^{9-}$ polyhedrons.

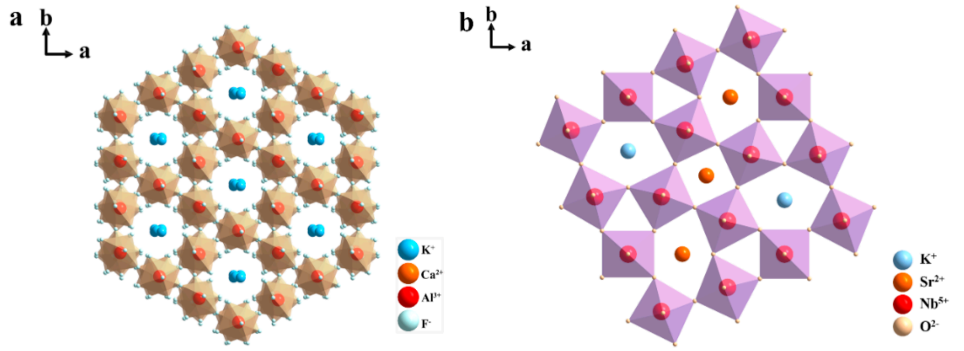

Figure S2. Crystal structure of **(a)** KCAF and **(b)** $Sr_2KNb_5O_{15}$ hosts from the [001] direction.

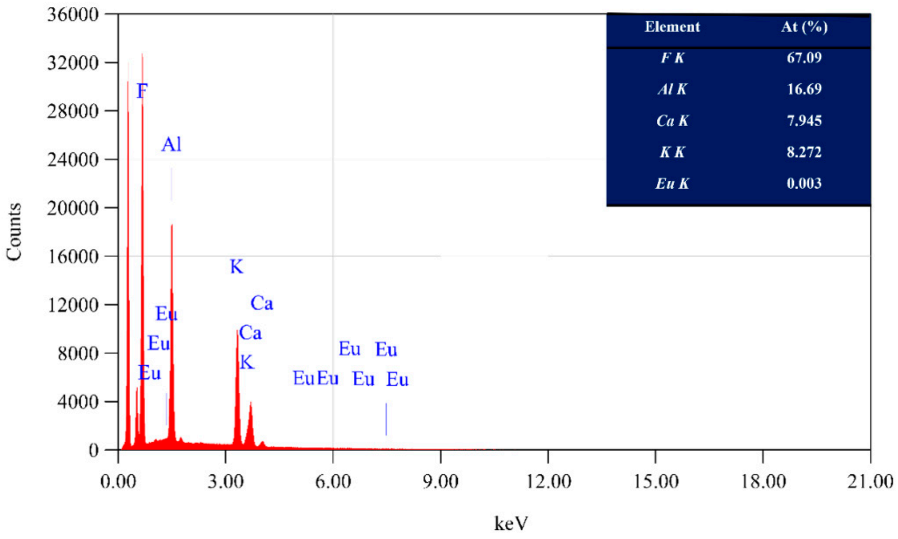

Figure S3. Energy-dispersive spectrum of KCAF:0.005Eu^{2+} phosphor, and the inset is the respective atom percentage of KCAF:0.005Eu^{2+}.

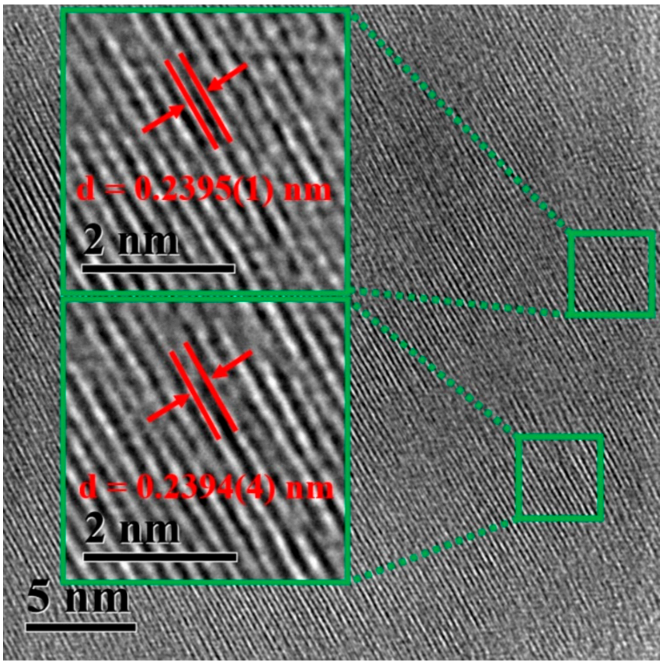

Figure S4. High-resolution TEM image of KCAF:0.005Eu^{2+}. The insets are the selected magnification areas in the corresponding high-resolution TEM image.

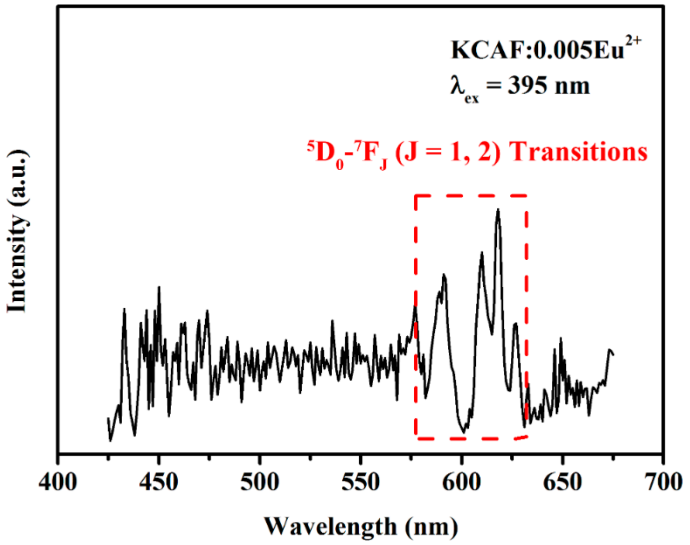

Figure S5. PL spectrum of KCAF:0.005Eu^{2+} phosphor (λ_{ex} = 395 nm).

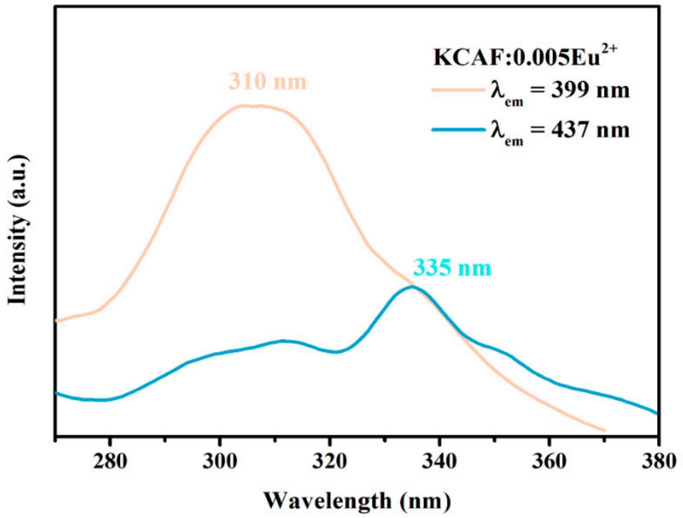

Figure S6. PLE spectra of KCAF:0.005Eu^{2+} phosphor monitored at 399 and 437 nm.

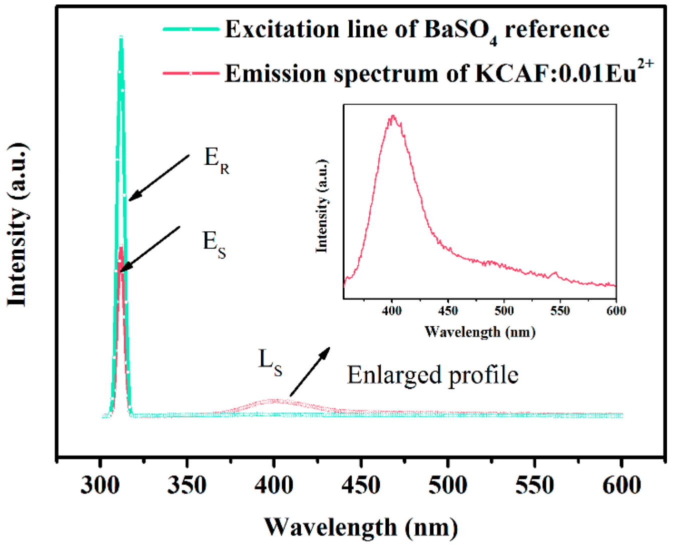

Figure S7. Excitation line of $BaSO_4$ and emission spectrum of the KCAF:0.01Eu^{2+} phosphor. The inset is the magnification of the emission spectrum.

[#] Note: In our work, the IQE, absorption efficiency (AE), and EQE values of the phosphor were calculated by the following equations:.

$$IQE = \frac{\int L_S}{\int E_R - \int E_S}$$

$$AE = \frac{\int E_R - \int E_S}{\int E_R}$$

$$EQE = IQE \times AE$$

where L_S is the luminescence emission spectrum of the sample; E_R and E_S are the spectrum of the excitation light without and with the measured sample in the integrating sphere, respectively.

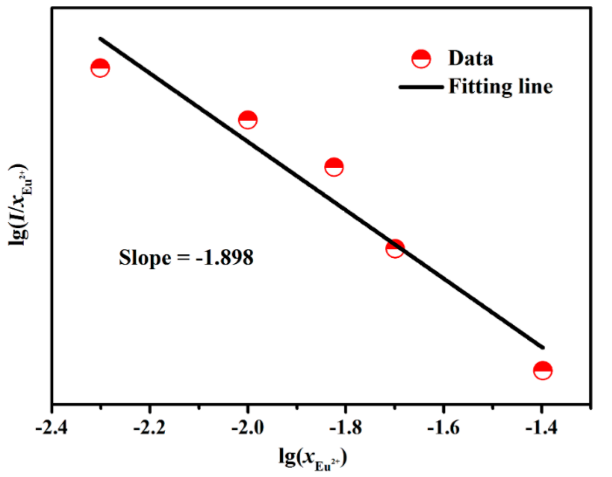

Figure S8. Line-fitting relationship of $\lg(I/x_{Eu^{2+}})$ versus $\lg(x_{Eu^{2+}})$.

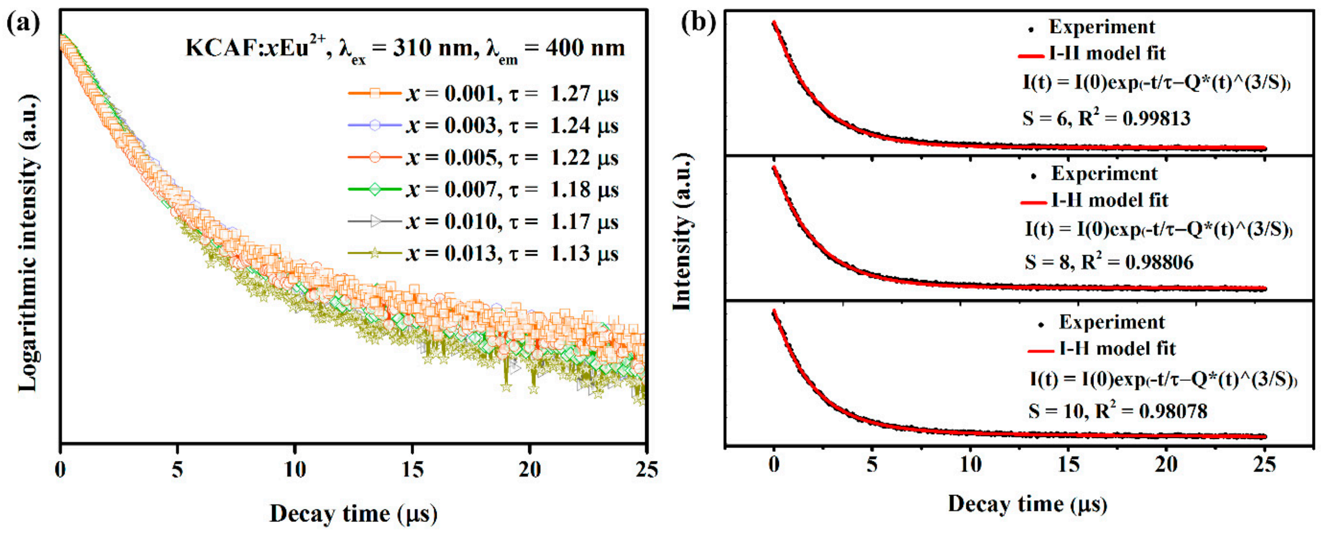

Figure S9. **(a)** The fluorescence decay curves of KCAF:xEu^{2+} (x = 0.001-0.013) phosphors with 310 nm excitation and 400 nm emission. **(b)** The fluorescence decay curves of KCAF:0.005Eu^{2+} phosphor. The solid lines correspond to the fits to the Inokuti-Hirayama model for S = 6, 8, and 10.

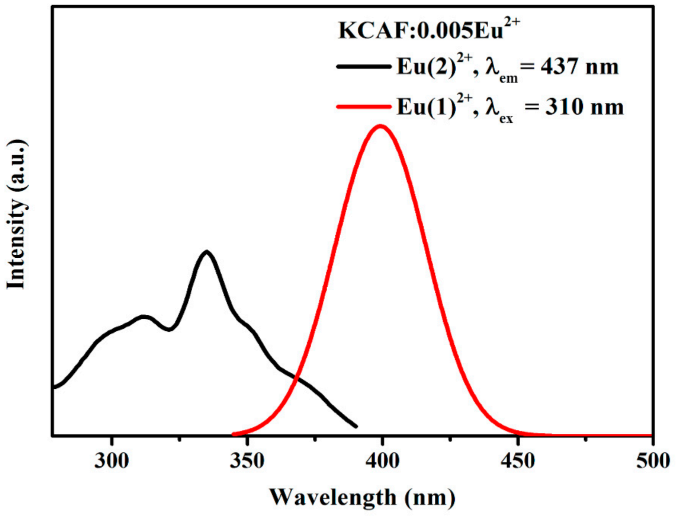

Figure S10. The PLE and PL spectra of KCAF:0.005Eu^{2+} phosphor (λ_{em} = 437 nm; λ_{ex} = 310 nm).

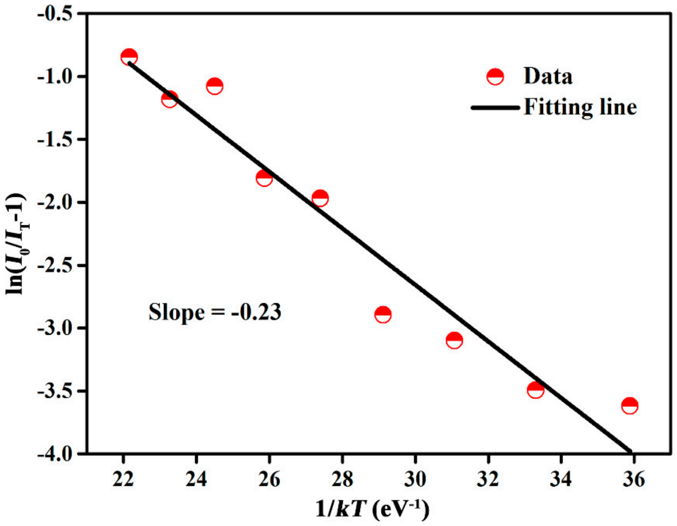

Figure S11. The activation energy for thermal quenching of KCAF:0.005Eu^{2+} phosphor.
